# About the usefulness of contact precautions for carriers of extended-spectrum beta-lactamase-producing *Escherichia coli*

**DOI:** 10.1186/s12879-015-1244-x

**Published:** 2015-11-12

**Authors:** Jean-Ralph Zahar, Laurent Poirel, Claire Dupont, Nicolas Fortineau, Xavier Nassif, Patrice Nordmann

**Affiliations:** Infection Control Unit, CHU Angers, Angers, France; Service de Microbiologie-Hygiène, Hôpital Necker-Enfants-Malades, Paris, France; Service de Bactériologie - Hygiène, Hôpital de Bicêtre, K.-Bicêtre, France; Department of Medicine, Medical and Molecular Microbiology « Emerging Antibiotic Resistance » Unit, Faculty of Science, University of Fribourg, Rue Albert Gockel 3, CH-1700 Fribourg, Switzerland; Hôpital Fribourgeois-hôpital Cantonal, Fribourg, Switzerland

**Keywords:** ß-lactamase, Infection control, Isolation, Hand hygiene

## Abstract

**Background:**

Extended-spectrum β-lactamases producing *Escherichia coli* (ESBL-E) are increasingly identified in health care facilities. As previously done for the control of methicillin-resistant *Staphylococcus aureus,* many hospitals have established screening strategies for early identification of patients being carriers of ESBL producers in general and ESBL-E in particular, and have implemented contact precautions (CP) for infected and colonized patients.

**Methods:**

The incidence of ESBL-E has been compared retrospectively between two French university hospitals (A and B) with different infection control policies over a 5-year long period of time (2006–2010).

**Results:**

While hospital A only implemented standard precautions after identification of patients colonized with ESBL-E, hospital B recommended additional CP. During the period of the study, the ESBL-E incidence rate significantly increased in both hospitals, but no significant difference was observed between the two hospitals.

**Conclusions:**

This observational study did not reveal that additional CP measures had a greater impact on the incidence of ESBL-E in hospital settings.

## Background

Since the early 21^th^ century, extended-spectrum ß-lactamase (ESBL) producing Enterobacteriaceae are spreading worldwide [1]. Those ESBL producers are mainly *Escherichia coli* isolates that may be found either in nosocomial settings but also in the community, and also *Klebsiella pneumoniae* and *Enterobacter cloacae* which are mainly nosocomial species. In some geographical areas, the rate of ESBL-producing *E. coli* is actually reaching 67 % [2]. Most of the ESBL enzymes are of CTX-M types, with CTX-M-15 being the most frequently identified variant in particularly in France [3]. The situation in France is endemic and the extensive spread of some specific clones such as *E. coli* ST131 have been shown to be a major feature [4]. However detailed molecular analyses of those ESBL producers showed some diversity in term of clones, isolates, plasmids, and ESBL genes [5]. Considering that the spread of ESBL-producing *E. coli* has reached an alarming rate, defining the most cost-efficient measures to control their spread in hospital settings represents a major issue.

In order to control and subsequently decrease the impact of ESBL producers in healthcare settings and related nosocomial infections, some authors [6] and medical societies actually recommend the implementation of contact precautions during hospitalization for colonized and infected patients, similarly to policies applied to control the spread of methicillin-resistant *Staphylococcus aureus* (MRSA).

To our knowledge, the most efficient control policies in reducing the incidence of ESBL-producing Enterobacteriaceae in hospital settings remain to be precisely determined. Many studies show that screening of ESBL producers at admission and implementing contact isolation might be effective for controlling their spread in hospital settings. However, most of those studies have been designed to control the spread of ESBL-producing *K. pneumonie* [7] or ESBL-producing *E. cloacae* and prevent related outbreaks that are quite commonly observed. By contrast, it is well admitted that nosocomial outbreaks linked to *E. coli* ESBL producers outbreaks are basically uncommon [8].

The aim of this study was to compare the incidence of ESBL-producing Enterobacteriaceae and specifically ESBL-producing *E. coli* in two French University hospitals located in the Paris area (Necker-Enfants-Malades hospital [hospital A] and Bicêtre hospital [hospital B]). In those two similar hospitals, distinct infection control policies regarding ESBL-producing *E. coli* were indeed implemented, making the comparison meaningful.

## Methods

### Setting

A retrospective study was conducted between January 1^st^ 2006 and September 30^th^ 2010 into all short-stay units from the hospitals A to B. Hospital A is a 550-bed University hospital with around 26,000 direct admissions per year, a mean hospitalisation stay of 5 days and a children/adult bed ratio of 4. Hospital B is an 800-bed University hospital with around 680 acute beds, almost 30,000 direct admissions per year, a mean hospitalisation stay of 7 days and a children/adult bed ratio of 0.2. In both hospitals all patients hospitalized in a short-stay unit were included as soon as a positive clinical sample growing an ESBL-producing Enterobacteriaceae was identified. Only the first ESBL-positive isolate was considered for each patient.

### Infection control policies

In both hospitals infection control policies for multidrug-resistant organisms (MDRO) consisted in active surveillance for patients hospitalized in intensive care units (rectal swab screening upon admission and weekly follow-up) and also in passive surveillance of all clinical samples tested routinely. Some specific measures could also be added in other hospitalization units in case of epidemic situations. When a MDRO was isolated (new patient detection or readmission of a known carrier) and required implementation of additional care precautions, the infection control team monitored health workers about the infection control recommendations (first by phone and then followed by a physical visit).

In France, the infection control guidelines currently suggest that, regardless of species, all patients infected or carriers of any type of ESBL-producing Enterobacteriaceae shall be isolated (http://www.sf2h.net/).

Contact isolation requires a private room, patient dedicated medical equipment and a specific signboard on the door of the patient’s room. Gown wearing is recommended when entering the patient room especially during a long and close care. Wearing gloves was mandatory when the health care worker was in contact with blood or body fluids. Hand hygiene involved hand rubbing with a waterless alcohol-based hand rub or hand washing with soap and water. As for standard care precautions, hand hygiene was recommended before and after touching the patient or his/her environment and after wearing gloves, i.e., after contact with blood or body fluids from the patient.

The hospital A Infection Control Committee decided to maintain strict contact isolation procedures for patients infected or colonized with any kind of ESBL producers, and the implementation and enforcement of the proposed measures was daily controlled. By contrast, isolation of patients infected or colonized with ESBL-producing *E. coli* was abandoned since 2008 at hospital B, with the exception of patients hospitalized in the neonatal intensive care unit. Noteworthy, contact isolation was maintained for all patients found to be infected or colonized by any other ESBL-producing Enterobacteriaceae. That decision resulted from the observation that the hospital was facing out a significantly increased incidence of ESBL-producing *E. coli.* That unexpected phenomenon which paradoxically did not result into an increased number of noscocomial outbreaks involving ESBL-producing *E. coli* in hospital B, nevertheless resulted in a major additional workload for the hospital staff. In fact, both hospitals applied the same infection control policies from 2006 to 2008 but adopted distinct policies during the 2008–2010 period.

Rectal swabs were plated onto selective chromogenic medium for screening of ESBL-producing Enterobacteriaceae (chromID ESBL Agar, bioMérieux, Marcy-l’Etoile, France). Bacteria were identified at the species level by standard biochemical-based techniques. Antibiotic susceptibility testing was performed and interpreted according to the EUCAST guidelines [9]. The double-disk synergy test was used to detect ESBL-producing Enterobacteriaceae.

### Hospital consumption data

Yearly volume of alcohol-based hand rub solution was defined as the total of volume consumption by hospital wards and expressed in liters by 1000 days of hospitalization. Antibiotic consumption was expressed as Defined Daily Dose according to WHO recommendation (http://www.whocc.no/atc_ddd_publications/guidelines/). These data were available from January 1^st^, 2006 to December 31^th^, 2009.

### Statistical analyses

Population characteristics were qualitatively described for each hospital in terms of medical activity, patients’ recruitment and ESBL-producing Enterobacteriaceae. Parametric (Chi2) or non parametric (Kolmogorov-Smirnov [KS]) tests were computed to quantify the difference between both hospitals on the basis of major descriptive factors, ie: age, gender, speciality (type of unit or clinical unit activity), clinical site and bacterial species.

Quarterly ESBL-producing Enterobacteriaceae incidence was defined as the number of clinical samples from which at least a single ESBL-producing Enterobacteriaceae had been isolated per patient, per 3 months and by 1000 days of hospitalization. The quarterly incidences were estimated for the main ESBL-producing enterobacterial species, namely *E. coli, K. pneumoniae* and *E. cloacae*. Evolution of those incidences were average plotted to observe trends and outbreaks. The difference in ESBL-producing *E. coli* incidence between hospitals A and B before and after 2008 was evaluated by a match-pairs *T*-test where each quarter represented a pair of data. Therefore, we could compare a difference of mean for eight quarters before the modification in hygiene practices in hospital B and a difference of mean for 11 quarters after 2008. Alcohol hand rub and antibiotics consumptions have been described by the average rate of increase or decrease of consumption per year between 2006 and 2009. All the statistics were performed using Excel Microsoft and SAS software (version 9.2, SAS Institute Inc., Carry, NC, USA).

## Results

Hospitals A and B were quite similar in terms of medical activity. The number of direct admissions per bed and year were respectively 47 (25, 882/551) in hospital A and 44 (29, 501/678) in hospital B despite a slight difference in the mean hospitalization stay (6.7 days for hospital B versus 5.4 days for hospital A).

A total of 499 and 659 clinical samples positive for ESBL-producing Enterobacteriaceae were included for hospitals A and B, respectively. As expected, the patients characteristics showed some significant differences between hospitals A and B, patients at hospital B being significantly older than patients at hospital A, median: 61 years-old versus 9 years-old (KS Test, *p* < 0.0001) but no significant difference was observed in the gender rate (Chi2 Test, *p* = 0.50).

Distribution of unit activity among each hospital was significantly different (Chi2 Test, *p* < 0.0001). More patients were hospitalized in intensive care units (ICUs) at hospital B whereas the proportions of patients from the medicine and surgery wards were quite similar for both hospitals, being respectively 53.4 to 41.1 % for hospital A, and 33.1 to 27.7 % for hospital B. When excluding patients hospitalized in ICUs, no significant difference was observed (Chi2 Test, *p* = 0.54).

Clinical sites associated with an ESBL-producing isolate were equally distributed between hospitals A and B except for the non-protected lower respiratory tract samples (10.2 % for hospital A versus 3.3 % for hospital B). The proportion of ESBL-producing *K. pneumoniae* was higher in hospital A compared to hospital B, being 37.5 and 27.5 %, respectively (Table 1).

**Table 1:** Features related to isolation of ESBL-producing Enterobacteriaceae in both hospitals during the Jan 2006 – Sept 2010 period

Features	Hospital A	Hospital B	*p*
Median age (Q1-Q3), *n*	9 (2–52), *n* = 499	61 (45–74), *n* = 659	<0.0001^*^
Gender			
Men	53.9 %, *n* = 269	51.9 %, *n* = 342	0.5
Women			
Hospitalization unit (%), *n*			
Medicine	53.4 %, *n* = 266	41.1 %, *n* = 271	<0.0001^*^
Surgery	33.1 %, *n* = 165	27.7 %, *n* = 183	without
ICU	13.5 %, *n* = 67	31.1 %, *n* = 205	ICU
Clinical site (%), n			0.54^**^
Urine	60.5 %, *n* = 302	59.9 %, *n* = 395	<0.0001^*^
Blood culture	10.2 %, *n* = 51	9.1 %, *n* = 60	without
Respiratory no protected	10.2 %, *n* = 51	3.3 %, *n* = 22	respiratory
Respiratory protected	3.8 %, *n* = 19	4.1 %, *n* = 27	no protected
Intravascular devices	4.7 %, *n* = 23	6.4 %, *n* = 42	0.07^**^
Deep purulent samples	1.8 %, *n* = 9	4.1 %, *n* = 27	
Others	8.8 %, *n* = 44	13.1 %, *n* = 86	
ESBL-producing Enterobacteriaceae by species (%), *n*			
*Escherichia coli*	38.7 %, *n* = 193	44.6 %, *n* = 294	0.0033^*^
*Enterobacter cloacae*	15.4 %, *n* = 77	19.3 %, *n* = 127	without *K. pneumoniae*
*Klebsiella pneumoniae*	37.5 %, *n* = 187	27.5 %, *n* = 181	0.73^**^
Other ESBL producers	8.4 %, *n* = 42	8.7 %, *n* = 57	

^*^ *p* < 0.05, or ^**^ *p* > 0.05 after deleting the varying category between hospital; Kolmogorov-Smirnov and Chi2 Test

The overall incidence rate of ESBL-producing Enterobacteriaceae significantly increased in both hospitals from January 2006 to September 2010, with a higher increase observed in hospital A compared to hospital B. These rates increased from 0.41 to 1.87/1000 patients-days in hospital A and from 0.54 to 1.31/1000 patients-days at hospital B.

**Fig. 1:**
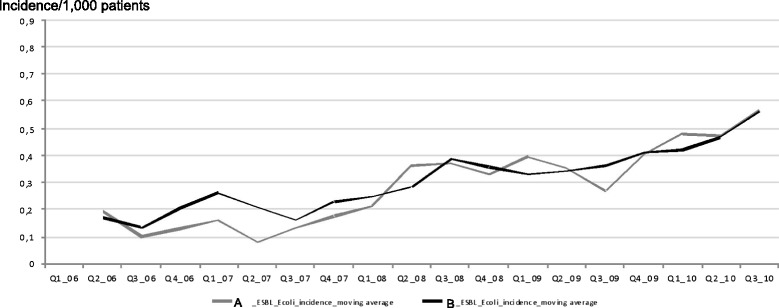
Respective quarterly ESBL-producing *E. coli* incidences in hospitals A and B; January 1^st^, 2006 to September 30^th^, 2010

**Fig 2:**
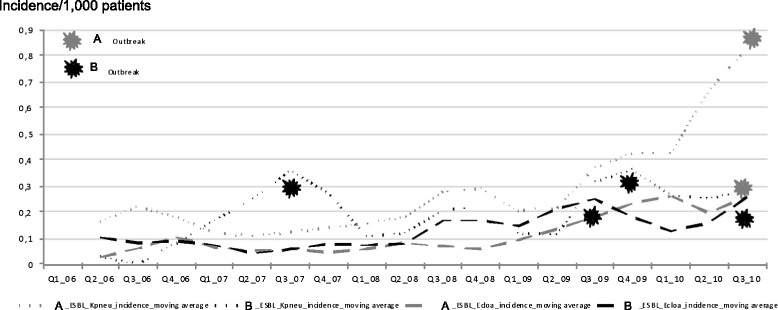
Respective quarterly ESBL-producing *K. pneumoniae* and *E. cloacae* incidences in hospitals A and B; January 1^st^, 2006 to September 30^th^, 2010

The evolution of quarterly incidences for ESBL-producing enterobacterial species differed when considered the three most prevalent species *E. coli*, *K. pneumoniae* and *E. cloacae*. Indeed, the rate of ESBL-producing *E. coli* increased linearly in both hospitals where no outbreak episode was observed during the study period, while the respective rates of ESBL-producing *K. pneumoniae* and ESBL-producing *E. cloacae* significantly increased and correlated with several outbreak episodes. In hospital B, four outbreak episodes occurred, corresponding to two outbreaks of infections caused by ESBL-producing *K. pneumoniae* in 2007 and 2009, and two outbreaks of infections caused by ESBL-producing *E. cloacae* in 2009 and 2010. In hospital A, two outbreak episodes occurred, corresponding to an outbreak of infections caused by ESBL-producing *E. cloacae* in 2009 and an outbreak of infections caused by ESBL-producing *K. pneumoniae* in 2010 (Figs. 1 and 2).

The impact of infection control policies was evaluated by comparing the respective incidences of ESBL-producing *E coli* in the two hospitals. Before 2008, the means of ESBL-producing *E. coli* quarterly incidences were respectively at 0.14/1000 patient-days (confidential interval [CI], 5 % [0.08–0.21]) in hospital A and 0.20/1000 patient-days (CI, 5 % [0.14–0.26]) in hospital B. After 2008, the means of ESBL-producing *E. coli* quarterly incidences were respectively 0.41/1000 patient-days (CI, 5 % [0.33–0.48]) in hospital A and 0.40/1000 patient-days (CI, 5 % [0.32–0.48]) in hospital B. Using a matched-paired *T*-test, the difference between quarterly incidences in hospital B minus hospital A (DeltaI compared to 0) were compared during the entire study period, but also before and after 2008 when new practices for management of patients infected or colonized by ESBL-producing *E. coli* were implemented in hospital B. When considering the entire study period, the DeltaI mean was 0.019/1000 patient-days, *p* = 0.36; before 2008, it was 0.059/1000 patient-days, *p* = 0.08; and after 2008, it was –0.009/1000 patient-days, *p* = 0.73. Therefore no difference was observed between the two hospitals in terms of incidence of ESBL-producing *E. coli* irrespective of the period of time (Table 2).

**Table 2:** ESBL-producing Enterobacteriaceae, isolated time (IT) by species, and considered variables^a^

	Median KS test (Q1-Q3), *p* ^b^					
Median IT with 1^st^ and 3^rd^ quartiles and Kolmogorov-Smirnov (KS) test	ESBL-positive *E. coli*		ESBL-positive *E. cloacae*		ESBL-positive *K. pneumoniae*	
Number of isolates (total = 1078)						
Hospital A	1.5 (0–9), *n* = 186		8.5 (2–23), *n* = 76		8 (1–23), *n* = 185	
		*0.13*		*0.009*		*0.04*
Hospital B	3 (0–13), *n* = 264		17 (7–31), *n* = 116		13 (3–28), *n* = 161	
Unit’s activity (*n* = 1077)						
Medicine (reference)	2 (0–8), *n* = 229		10.5 (2–31), *n* = 74		8 (1–23), *n* = 161	
Surgery	2.5 (0–14), *n* = 152	*0.30*	13 (5–24), *n* = 54	*0.66*	7 (1–17), *n* = 85	*0.67*
ICU	5 (1.5–13.5), *n* = 68	*0.04*	15 (7.5–28.5), *n* = 64	*0.07*	17.5 (7.5–37), *n* = 100	*0.003*
Age (*n* = 1058)						
Children (<14 y-old)	2.5 (0–9.5), *n* = 120		7 (1–22), *n* = 37		8 (2–26.5), *n* = 136	
		*0.97*		*0.08*		*0.61*
Adult	2 (0–11), *n* = 318		14 (6–27), *n* = 153		11 (1–24), *n* = 148	
Gender (*n* = 1078)						
Men	4 (0–15), *n* = 199		14 (5–28.5), *n* = 112		10 (1–24), *n* = 198	
		*0.09*		*0.87*		*0.61*
Women	2 (0–8), *n* = 251		14 (5–27), *n* = 80		9 (1–25.5), *n* = 148	
Clinical site (*n* = 961)						
Urine (reference)	2 (0–10.5), *n* = 316		14 (4–28.5), *n* = 96		8 (1–23), *n* = 181	
Blood culture	2 (0–7), *n* = 37	*0.67*	15.5 (5–28), *n* = 26	*0.99*	11 (1–28), *n* = 33	*0.41*
Respiratory protected	5 (1–14), *n* = 11	*0.94*	12 (5.5–24), *n* = 12	*0.63*	19 (5–62), *n* = 19	*0.10*
Respiratory unprotected	2 (1–7), *n* = 12	*0.96*	13 (2–26), *n* = 14	*0.86*	8.5 (3–21), *n* = 38	*0.06*
Intravascular devices	8 (4–9), *n* = 7	*0.05*	14 (7–25), *n* = 21	*0.18*	14 (9–21), *n* = 27	*0.006*
Deep purulent samples	17 (1–41), *n* = 17	*0.007*	18 (4–27), *n* = 7	*0.99*	19 (14–21), *n* = 6	*0.19*
Total by ESBL producer	2 (0–11), *n* = 450		14 (5–28), *n* = 192		10 (1–24), *n* = 346	

^a^Total numbers may differ according to categories since some data were not always available; ^b^significant level, *p* < 0.05

The consumption of alcohol-based hand rubs (AHRs) and antibiotics was similar during the 2006–2009 period in both hospitals, with an increase of AHR (+20 %/year for hospital A and +28 %/year for hospital B) and a decrease of antibiotic consumption (−4 %/year for hospital A and −3 %/year for hospital B).

## Discussion

During this retrospective study performed in two University hospitals in the Paris area, an increased isolation of ESBL-producing Enterobacteriaceae from infected sites was observed from 2006 to 2010. The increase was mainly related to the increased incidence of ESBL-producing *E. coli*, which is basically related to its increased prevalence in the community [10]. However, it is noteworthy that no entry screening was performed and therefore the true burden of patients being already colonized with ESBL-producing *E. coli* at admission remains unknown in our study. In addition, no molecular typing was performed here and we cannot completely assess that ESBL-producing *E. coli* isolates were transmitted or just brought by the patients. These are two important features that might be considered in further studies. Interestingly, despite strict adherence to national guidelines, isolation policies adopted in the two hospitals from 2006 to 2008 were not associated with a decreased incidence of ESBL-producing *E coli*. Moreover no difference was observed between the two hospitals despite discontinuation of the isolation policy in hospital B after 2008. This result contradicts studies indicating the efficacy of these measures (together with other interventions) to control the spread of different resistant bacteria such as MRSA [11] vancomycin-resistant *Enterococci* [12] and ESBL-producing *K. pneumoniae* [7], and from which is is believed that CP does prevent cross transmission*.*

These data suggest that contact isolation procedures aimed to prevent or to limit the spread of ESBL-producing *E. coli* are actually inefficient. This might be explained by the fact that contact isolation might be poorly beneficial in a context where health care workers hand hygiene compliance is either high or very low [13]. Another issue is that contact isolation has almost no role in preventing endogeneous infections caused by the patient’s gut flora, which has to be placed into the perspective of an increased prevalence of ESBL-positive *E. coli* in the community in France [14].

Other hypotheses might explain the lack of efficiency of contact isolation for controlling the spread of ESBL-producing *E. coli.* Indeed, the spread of MDRO depends on numbers of factors such as colonization pressure (i.e., number of patients admitted with MDRO), cross transmission (i.e., adherence to hand hygiene), and selective pressure (i.e., antibiotic consumption) [15, 16]. Considering the high rate of ESBL-producing *E coli* in the community and the number of risk factors associated with such carriage, it seems very challenging to identify patients carrying ESBL-producing *E. coli* upon admission. In fact, according to several recent publications [17], only 15 % of patients colonized with ESBL-producing *E. coli* actually develop a clinical infection related to that organism. Noteworthy, a study performed with patients with leukemia or haematopoietic stem cell transplantation could not confirm any association between colonization and infection with ESBL-producing *E. coli* or any increased hospital mortality [18]. Therefore, limiting isolation policies to infected patients only might have a limited impact by missing a large proportion of carriers. It is known that infection control measures implemented to control the spread of MRSA strains must include infected but also colonized patients to be efficient [19].

The increased rate of ESBL-producing *E. coli* in the two hospitals could also be explained by a non-effective antibiotic stewardship, despite adherence to a national program implemented since 2005 that resulted into a decrease of antibiotic consumption by 10 % in hospital B and by 12.5 % in hospital A between 2006 and 2009. Indeed, several studies have shown the beneficial outcome of antibiotic usage restriction in decreasing the incidence of ESBL-producing Enterobacteriaceae [7].

One of the major issue raised by this study is the problem to evaluate the risk of bacterial spread at the species level. Analysis of the literature shows that very few nosocomial outbreaks of ESBL-producing *E. coli* actually occur and that those which have been described occur in special hospital conditions such as neonates or long term care facilities [20]. Hence most of the hospital-based outbreaks involving ESBL producers are not caused by *E. coli* but by *K. pneumoniae*. Very few (if any) data actually exist to assess differential risks between bacterial species. To our knowledge there is no study comparing environmental persistence of ESBL-producing *E. coli* and ESBL-producing *K. pneumoniae* or *E. cloacae.*

Our study basically has several limitations. First, we were unable to identify patients carrying ESBL-producing *E. coli* at admission and to define the specific colonization rate in each hospital. However, since both hospitals are located in the same geographical area, we may hypothetize that the prevalence of patients colonized by ESBL-producing Enterobacteriaceae at admission were similar. Then, considering that antibiotic selective pressure likely plays a role in the spread of ESBL-producing Enterobacteriaceae in hospitals, one might argue that the difference of antibiotic consumption could explain our results. Also, it is clear that the two hospitals do not recruit the same types of patients, and median ages have been estimated to be significantly different (61 vs 9 years-old). Hospital A possesses more pediatric wards than hospital B, therefore one could argue that the risk of cross transmission in those wards could be higher than in adult wards. We might also consider that the incidences of ESBL producers are significantly different.

Finally, since active surveillance at admission was not implemented except for the ICU, we cannot formally assess that positive cases were indeed hospital-acquired.

## Conclusion

In conclusion, no significant difference between incidences of ESBL-producing *E. coli* were noted between the two hospitals, regardless of the different policies of patient isolation followed. Considering that infection control activity is costly and time-consuming, and also that the reservoir of ESBL-producing *E. coli* is increasing worldwide, we believe all efforts should focus on all ESBL-producing Enterobacteriaceae other than *E. coli* to prevent nosocomial outbreaks. It is important to highlight that the findings we actually observed may not be generalizable to other settings, especially neonatal wards, neonatal intensive care units, or nursing homes, where outbreaks of ESBL-producing *E. coli* have been described [21–25].

Since nosocomial outbreaks caused by ESBL-producing *K. pneumoniae* are frequent, a meaningful strategy would be to early identify patients being colonized with ESBL-producing *K. pneumoniae*, differentiating them from patients colonized by ESBL-producing *E. coli*. Nowadays this can easily be done using selective chromogenic culture media followed by rapid identification of ESBL producers [26].

### Ethics statement

This study was retrospective. Retrospective studies and their corresponding data do not need ethics approval in France.

## Abbreviations

AHRs: Alcohol-based hand rubs
CI: Confidential interval
ESBL: Extended-spectrum ß-lactamase
ICU: Intensive care unit
KS: Kolmogorov-Smirnov
MRSA: Methicillin resistant *Staphylococcus aureus*
MDRO: Multidrug resistant organisms
